# Myocardial Bridge of the Left Anterior Descending Artery Causing Pseudo-Wellens’ Syndrome: A Report of Two Cases

**DOI:** 10.5811/cpcem.1404

**Published:** 2023-05-26

**Authors:** Debayan Guha, Franz C. Mendoza-Garcia, Kathryn M. Millen, Joseph Offenbacher, Nicholus M. Warstadt

**Affiliations:** * Albert Einstein College of Medicine, Department of Emergency Medicine at the Jacobi and Montefiore Medical Centers, Bronx, New York; † New York University Grossman School of Medicine, Ronald O. Perelman Department of Emergency Medicine, New York, New York

**Keywords:** emergency medicine, acute coronary syndrome, Wellens’ syndrome, myocardial bridge, case report

## Abstract

**Introduction:**

Wellens’ syndrome represents an important, at times overlooked, spectrum of left anterior descending (LAD) coronary artery occlusion, spontaneous reperfusion, and impending reocclusion. Once considered pathognomonic for a thromboembolic coronary event, an increasing number of clinical scenarios have been demonstrated to result in pseudo-Wellens’ syndrome, each requiring unique forms of assessment and management.

**Case Report:**

We describe two clinical presentations in which myocardial bridging (MB) of the LAD led to clinical and electrophysiologic presentations of a pseudo-Wellens’ syndrome.

**Conclusion:**

These reports represent a rare cause of pseudo-Wellens’ syndrome attributed to MB of the LAD. Transient ischemia secondary to myocardial compression of the traversing LAD leads to intermittent angina and electrocardiogram changes that are typical in patients presenting with Wellens’ syndrome secondary to an occlusive coronary event. As with other previously reported pathophysiologic mechanisms that have been shown to mimic Wellens’ syndrome, myocardial bridging should be considered in patients presenting with a pseudo-Wellens’ syndrome.

## INTRODUCTION

First described in 1982, Wellens’ syndrome represents an important cardiovascular syndrome in the emergency department (ED). Defined by its electrocardiogram (ECG) findings in the context of waxing and waning angina, Wellens’ syndrome was historically taught to herald impending cardiac ischemia due to a left anterior descending (LAD) thromboembolic lesion.^1^ It is now understood to represent an evolving pathophysiologic pattern of occlusion and spontaneous reperfusion of the LAD. The term pseudo-Wellens’ syndrome is used when the etiology of the patient’s Wellens’-like ECG pattern is not due to an atherosclerotic event.

Myocardial bridging (MB) is a congenital variant in which a coronary artery tunnels through the myocardium and the tunneled artery is constricted on ventricular systole.^2^ We describe two cases of a pseudo-Wellens’ syndrome attributed to MB of the LAD. We hope to highlight the rapid identification of Wellens’ syndrome via clinical presentation and ECG pattern, educate emergency physicians on MB as a possible anginal cause, and further describe a rare clinical entity.

## CASE REPORTS

### Case One

A 67-year-old female with a past medical history of diabetes, hypertension, hypothyroidism, scoliosis, spinal stenosis, chronic back pain, and cervical radiculopathy presented to the ED complaining of atraumatic neck and back pain radiating to her left shoulder. She denied chest pain, shortness of breath, or any other active cardiopulmonary symptoms. Her pain was not exertional, pleuritic, or positional. Her review of systems was otherwise negative.

On presentation to the ED, her heart rate was 78 beats per minute, blood pressure was 143/62 millimeters of mercury (mm Hg), respiratory rate 14 breaths per minute, oxygen saturation s 99% on room air, and temperature was 36.7° Celsius. She was well-appearing in no acute distress, and there were no notable findings on cardiopulmonary, neurologic, or musculoskeletal exams. Her ECGs are summarized in Images 1A and 1B.

Laboratory studies were significant for mild anemia with a hemoglobin of 11.4 grams per deciliter (g/dL) (reference range: 11.9–15.3 g/dL). Serum troponin and creatinine phosphokinase were within normal limits. Chest radiograph showed a prominent heart size and calcified aorta with clear lungs. Cardiology was consulted for possible Wellens’ syndrome and were concerned for a proximal LAD lesion. The patient received 325 milligrams (mg) aspirin and was started on intravenous (IV) heparin. She was taken for urgent coronary catheterization, which showed a patent coronary lumen. A MB in the mid-LAD was discovered and was thought to explain the patient’s clinical presentation and ECG findings (Images 2A and 2B).

*CPC-EM Capsule*

What do we already know about this clinical entity?

*Wellens’ Syndrome is considered an indicator of impending left anterior descending occlusion. Non-thromboembolic mimics have been termed pseudo-Wellens’*.

What makes this presentation of disease reportable?

*Myocardial bridging is a rare cause of pseudo-Wellens’. Here, two patients with variable presentations are described*.

What is the major learning point?

*It is important to recognize various pathophysiological mechanisms for pseudo-Wellens’ as treatment may differ from treatment of an acute coronary syndrome*.

How might this improve emergency medicine practice?

*This report adds to the knowledge of pseudo-Wellens’ syndromes, describes physiology of myocardial bridging, and discusses management of patients with known MB*.

### Case Two

A 56-year-old female with a past medical history of hypertension and hyperlipidemia presented to the ED with a two-day history of exertional, left-sided chest pain and dyspnea. Chest pain was non-radiating, described as a tightness, and 10/10 in severity when present. She had associated lightheadedness. She denied symptoms at rest and was asymptomatic while in the ED. Review of systems was otherwise negative. On presentation to the ED, her heart rate was 86 beats per minute, blood pressure was 137/89 mm Hg, respiratory rate 16 breaths per minute, oxygen saturation 98% on room air, and temperature was 36.6° Celsius. Her physical exam was unremarkable.

Her initial ECG, concerning for Wellens’ syndrome, is shown in Image 3A. An ST-elevation myocardial infarction (STEMI) alert was activated, the patient was given 325 mg of aspirin, started on IV heparin, and taken for cardiac catheterization. Blood laboratory analysis sent prior to the catheterization were remarkable for a troponin I concentration of 0.37 nanograms per milliliter (ng/mL) (reference range: < 0.04 ng/mL). Complete blood count, complete metabolic panel, coagulation studies, lipid panel, and hemoglobin A1c were unremarkable.

The patient’s left heart catheterization showed mild luminal irregularities in the LAD without a culprit lesion. A mid-LAD MB was identified. No percutaneous coronary intervention (PCI) was performed. Subsequent transthoracic echocardiogram and cardiac magnetic resonance imaging were unremarkable. The patient was started on aspirin and metoprolol, and she was discharged on hospital day two.

Two weeks after her hospital discharge and while in the cardiology office for follow-up, the patient experienced a similar episode of 10/10 chest pain. Her ECG is shown in Image 3B. The patient was transferred to the ED, where she reported ongoing chest pain. Her heart rate was 77 beats per minute, blood pressure 121/84 mm Hg, respiratory rate 16 breaths per minute, oxygen saturation 100% on room air, and temperature was 36.6° Celsius. She appeared uncomfortable with an otherwise unremarkable physical exam.

Laboratory analysis was notable for a troponin I concentration of 0.37 ng/mL. Due to ongoing chest pain with new ECG changes, the patient went for coronary angiography, which showed the known MB without new plaque rupture, occlusion, dissection, or alternate explanation for pain. During her hospitalization, metoprolol was discontinued, and verapamil was initiated. She was discharged on hospital day two with cardiology and cardiac surgery follow-up. Despite medical therapy, the patient remained symptomatic at follow-up. She was not interested in surgical intervention.

## DISCUSSION

Wellens’ syndrome is an important clinical entity in the ED suggesting a dynamic occlusion, reperfusion, and impending reocclusion of the LAD. Once considered to be a STEMI equivalent, evolving understanding of this syndrome calls for aggressive medical management and urgent coronary catheterization.

Traditionally, the syndrome was thought to consist of two distinct ECG patterns found in patients with a history of unstable angina. Type A (25% of patients) presents with biphasic T-waves in V2–V3. Type B (75% of patients) presents with deep symmetric T-wave inversions in V2–V3.^3^ These findings represent critically high grade LAD stenosis with a high specificity of 96.2%.^4^ While patients are often pain-free at the time the ECG is taken, subsequent anterior wall myocardial infarction is likely.

While Wellens’ syndrome is associated with thromboembolic occlusion of the LAD, several other physiologic mechanisms have been shown to mimic this electrocardiographic presentation due to transient obstruction of coronary flow and are categorized as pseudo-Wellens’ syndrome. Coronary vasospasm from cocaine use,^5^ pulmonary embolism,^6^ stress cardiomyopathy,^7^ and uncontrolled hypertension^8^ have been reported to cause pseudo-Wellens’ syndrome.

Myocardial bridging describes a congenital variant characterized by an intramyocardial route of a segment of one of the major coronary arteries, generally the LAD. The rate of MB in the general population ranges from 5–40%.^2^ Often discovered incidentally, prevalence is impacted by the diagnostic modality used (coronary angiography, computed tomography, etc). Patients with hypertrophic cardiomyopathy have exponentially higher incidence.^9^ Myocardial bridging of the LAD is associated with myocardial ischemia, the development of dysrhythmias, and sudden cardiac death.^2^ Limited evidence suggests the use of beta-blockers (BB) or non-dihydropyridine calcium-channel blockers (CCB) as first-line medical therapy. Stenting is controversial due to high rates of revascularization. If medical management fails, myotomy and coronary artery bypass grafting are viable surgical options. Nitroglycerin accentuates systolic compression of bridged segments and is contraindicated.^2^ Exercise may induce symptoms and has even been reported to cause fatal arrythmia,^10^ although no consensus guidelines exist for exercise abstinence.

The typical angiographic feature of a MB is systolic narrowing of a coronary artery, which often resolves completely during the diastolic phase of the cardiac cycle. Because only 15% of coronary flow normally occurs during systole and MB is a systolic angiographic event, clinically significant ischemia has only been demonstrated in specific clinical scenarios. Most commonly, tachycardia can provoke an ischemic event secondary to MB due to the shortening of the diastolic phase and the subsequent increased significance of systolic coronary perfusion.^11^ If the myocardial muscle is hypertrophic, the tunneled artery can be compressed during each cycle of systole.^12^

Rarely, MB has been reported to cause pseudo-Wellens’ syndrome.^12^–^15^ In each case previously described, the patient presented with classic anginal symptoms and a Wellens’- pattern ECG. Each patient had negative serum troponin concentrations. All patients responded to medical therapy, which primarily included BB, CCB, aspirin, and clopidogrel; and none required PCI or surgery.

In both cases presented here, MB of the mid-LAD, diagnosed via coronary angiography, caused transient LAD stenosis, resulting in pseudo-Wellens’ syndrome. Case one is unique in that the patient had neither chest pain nor classic anginal symptoms. Nevertheless, her dynamic ECG changes were concerning for ischemia, which prompted coronary angiography and led to the diagnosis of MB. Case two is the first in the literature to describe pseudo-Wellens’ from MB leading to a positive troponin and in which the patient had early treatment failure to pharmacological interventions.

## CONCLUSION

Myocardial bridging should be recognized as a possible etiology of pseudo-Wellens’ syndrome. Because Wellens’ syndrome often predicts imminent critical ischemia and reports of pseudo-Wellens’ syndromes are rare, acute coronary syndrome should be empirically treated and appropriately ruled out in patients presenting with a concerning history and a Wellens’-pattern ECG. Symptomatic worsening following nitroglycerin administration should increase suspicion for MB. Diagnosis is made by coronary angiography or non-invasive advanced coronary imaging techniques. After diagnosis is made, medical management typically includes initiation of beta blockers or calcium-channel blockers, with surgery reserved for refractory cases. Percutaneous coronary intervention is controversial. Patients should be followed by cardiology and cardiac surgery.

## Figures and Tables

**Image 1A:**
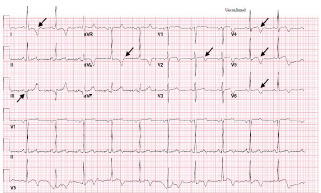
Electrocardiogram on presentation with Q wave in III, deep T-wave inversions in leads I and aVL, and biphasic T-wave morphology in leads V2 and V4–V6; annotated with black arrows.

**Image 1B:**
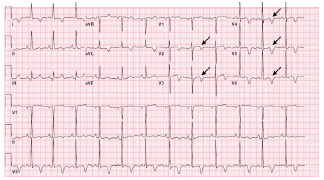
Follow-up electrocardiogram 20 minutes after presentation with persistent biphasic T-wave inversion in V2, evolving biphasic T-wave morphology in V3, and deeply symmetrically inverted T-waves in V4–V6; annotated with black arrows.

**Image 2A:**
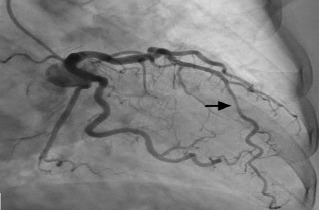
Coronary angiogram demonstrating mid-left anterior descending coronary artery occlusion during systole (arrow).

**Image 2B:**
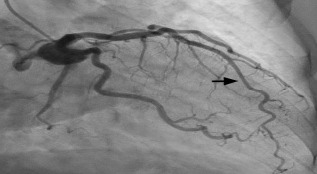
Coronary angiogram demonstrating mid-left anterior descending patency and distal coronary artery reperfusion during diastole (arrow).

**Image 3A:**
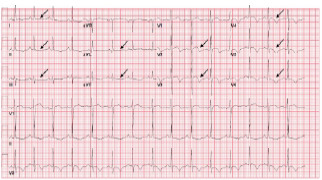
Presenting electrocardiogram showing T-wave flattening in leads I and aVL; subtle T-wave inversions in II, III, and aVF; new biphasic T-waves in V2 and V3; and new deep symmetric T-wave inversions in V4–V6; annotated with black arrows.

**Image 3B:**
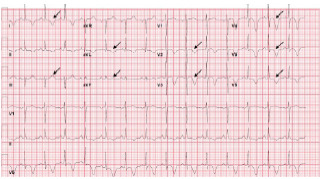
Electrocardiogram on second presentation showing new T-wave inversions in leads I and aVL; pseudo-normalization of T-waves in III and aVF; and deepening of previously noted T-wave inversions in V2–V6; annotated with black arrows.
